# Alterations in sulcal depth and associated functional connectivity in schizophrenia with auditory verbal hallucinations

**DOI:** 10.3389/fpsyt.2025.1641190

**Published:** 2025-07-30

**Authors:** Zhenru Guo, Zimo Zhou, Shuai Wang, Lianlian Yang, Xiaoshan Gao, Yu Xia, Yuanyuan Yang, Zhangyan Shan, Haixia Huang, Lin Tian

**Affiliations:** ^1^ Department of General Psychiatry, The Affiliated Mental Health Center of Jiangnan University, Wuxi, Jiangsu, China; ^2^ School of Wuxi Medicine, Nanjing Medical University, Wuxi, Jiangsu, China; ^3^ Department of Medical Imaging, Shanghai Health and Medical Center, Wuxi, Jiangsu, China

**Keywords:** schizophrenia, auditory verbal hallucinations, sulcal depth, functional connectivity, default mode network, visual cortex

## Abstract

**Background:**

Schizophrenia patients with auditory verbal hallucinations exhibit brain structure abnormalities. However, the characterization of sulcal depth alterations and associated functional connectivity across the whole brain remains unclear.

**Method:**

We recruited 38 schizophrenia patients with auditory verbal hallucinations and 31 schizophrenia patients without auditory verbal hallucinations. Magnetic resonance imaging data were collected on all participants, and clinical symptoms were assessed using standardized clinical scales. Structural abnormalities identified through sulcal depth analysis were localized to specific brain regions, which were subsequently selected as seed regions for functional connectivity analysis. Correlation analysis was employed to explore the associations between sulcal depth, functional connectivity, and the severity of clinical symptoms in individuals with schizophrenia with auditory verbal hallucinations.

**Results:**

Schizophrenia patients with auditory verbal hallucinations exhibited significantly increased sulcal depth in left hemispheric regions including the lingual gyrus, cingulate gyrus, pericalcarine cortex, parahippocampal gyrus, superior frontal gyrus, cuneus, and precuneus, whereas decreased sulcal depth was observed in right hemispheric regions encompassing the superior parietal gyrus, superior frontal gyrus, lingual gyrus, lateral occipital cortex, fusiform gyrus, postcentral gyrus, middle frontal gyrus, precentral gyrus, inferior temporal gyrus, precuneus, and parahippocampal gyrus compared to schizophrenia patients without auditory verbal hallucinations. Seed-based functional connectivity analysis revealed widespread weakened connectivity in schizophrenia patients with auditory verbal hallucinations, particularly with the superior frontal gyrus, angular gyrus, putamen, and other regions. The increased sulcal depth cluster in schizophrenia patients with auditory verbal hallucinations was significantly correlated with negative syndromes and general psychopathology of Positive and Negative Syndrome Scale.

**Conclusion:**

These findings highlight sulcal depth and associated functional connectivity abnormalities in schizophrenia patients with auditory verbal hallucinations, implicating early neurodevelopmental disturbances involving the default mode network and visual cortex. Sulcal depth may represent a promising biomarker for early diagnosis.

## 1 Introduction

Schizophrenia (SCZ) is a severe, chronic psychiatric disorder characterized by heterogeneous symptoms, including hallucinations, delusions, disorganized thinking, and affective flattening (1). Among these, auditory verbal hallucinations (AVH), defined as the perception of speech in the absence of external auditory stimuli, represent one of the most prevalent and clinically significant positive symptoms, affecting approximately 60–80% of individuals with SCZ (2, 3). These hallucinations frequently consist of negative or commanding content, which can cause substantial distress and functional impairment (4). AVH profoundly affects patients’ quality of life and is closely associated with increased risks of self-harm and suicide, as well as poorer long-term clinical outcomes (5). Despite the availability of various treatment approaches, including antipsychotic medications, brain stimulation techniques, and cognitive behavioral therapy, a considerable proportion of patients (around 30%) continue to experience persistent AVH that is resistant to conventional interventions (6). The persistence and severity of AVH in SCZ highlight a critical need to better understand their underlying neurobiological mechanisms.

Growing evidence suggested that AVH is linked to impairments in the brain’s auditory processing pathways (7, 8). Structural Magnetic Resonance Imaging (MRI) revealed consistent gray matter volume (GMV) reductions in key regions of SCZ patients with AVH, notably the left anterior insula and inferior frontal gyrus (9), with inverse correlations between AVH severity and GMV in the fusiform gyrus, inferior temporal gyrus (ITG), orbitofrontal gyrus, and superior frontal gyrus (SFG) (10). While GMV reflects broad neurodevelopmental changes, surface-based morphological analyses reveal more detailed cortical abnormalities, such as cortical thickness and gyrification index (11). Multiple neuroimaging studies identified reduced cortical thickness in SCZ patients with AVH, particularly in the temporal lobe and orbitofrontal cortex regions (12, 13). However, SCZ patients with AVH exhibit increased gyrification index specifically in the left superior parietal gyrus (SPG) and right anterior cingulate cortex (14). These diverse morphological observations highlight the need for more refined and in-depth investigations into cortical architecture, particularly through advanced surface-based metrics that can capture subtle structural variations beyond conventional measures.

Sulcal depth is a crucial morphological feature that reflects cortical complexity and folding patterns. Unlike other cortical measures, sulcal depth demonstrates remarkable neurodevelopmental stability, forming during fetal and infant stages before stabilizing in childhood and adolescence (15). Critically, this stability persists throughout the lifespan, a recent large-scale review confirming sulcal pits as fixed neuroanatomical markers (16). Notably, their integrity is maintained even in elderly populations, despite accelerated cortical atrophy typically emerging at the late age of 70 (17). Emerging evidence suggested that interindividual variations in sulcal depth correlate with cognitive function, intelligence, and neuropsychiatric disorders (18–20). Despite its potential significance, research on sulcal depth in SCZ patients with AVH remains limited, with only two studies examining sulcal pits to date. The first study (21) reported that the distribution of sulcal pits in the left superior temporal gyrus of SCZ patients with AVH was less than healthy controls, suggesting that the patients had an atypical morphological pattern. Another study (22) revealed that SCZ patients with AVH exhibited increased sulcal depth in the left inferior frontal cortex and prefrontal regions compared to SCZ patients who had never experienced AVH, which overlapped with Broca’s area and Brodmann area 47. Comparisons between SCZ patients with AVH and healthy controls revealed nearly identical results. These results indicated that early-emerging morphological abnormalities in language-related cortical areas may contribute to AVH vulnerability in SCZ.

Previous studies suggested that the structural characteristics of the cerebral cortex may influence brain function (23, 24). Abnormal cortical folding can alter the local neural circuit topology (25) and may disrupt the local microcircuit excitation and inhibition balance (26), leading to abnormal information output in specific brain regions, which can then spread through long-range connections to the whole-brain network, causing functional connectivity (FC) abnormalities. A recent study further supports this notion by showing that impaired corollary discharge and efference copy signals due to abnormal prefrontal-motor cortex connectivity lead to misinterpretation of self-generated speech as external voices, contributing to AVH (27). However, current studies focus on a single modality and selectively analyze brain regions, which fail to comprehensively capture the characteristics of sulcal depth across the whole brain. Importantly, the functional implications of abnormal sulcal depth remain largely unexplored. Addressing this critical knowledge gap is essential for advancing the development of more effective and targeted interventions to alleviate this debilitating symptom. Given that sulcal depth reflects cortical folding patterns and may index region-specific neurodevelopmental abnormalities, we hypothesized that structural deviations in sulcal depth would correspond to alterations in functional integration across neural circuits. To test this hypothesis, we conducted a comprehensive multimodal neuroimaging study combining whole-brain structural and functional analyses using seed-based FC analysis. First, we aimed to confirm whether SCZ patients with AVH demonstrate sulcal depth alterations consistent with previous findings. Subsequently, we examined whether corresponding FC abnormalities were present in these regions. Finally, we investigated relationships between these neuroimaging changes and clinical symptoms.

## 2 Materials and methods

### 2.1 Participants

All participants were recruited from the Mental Health Centre of Jiangnan University, China. The participants consisted of 38 SCZ patients with AVH (AVH patients) and 31 SCZ patients without AVH (NAVH patients). The study enrolled Han Chinese participants aged 18–65 years who were right-handed and had normal hearing. All patients were diagnosed with SCZ by senior psychiatrists in accordance with DSM-5 criteria (28). AVH patients were required to have a score ≥ 4 on the Positive and Negative Syndrome Scale (PANSS) P3 hallucination item, with clinical confirmation of AVH (29), while NAVH patients were required by a score = 1 on the PANSS P3 item hallucination and absence of all hallucination subtypes. Exclusion criteria included other psychiatric disorders (e.g., schizoaffective disorder, mood disorders, dementia, or substance dependence), severe or unstable somatic diseases (e.g., heart disease), narrow-angle glaucoma, a history of epilepsy or neuroleptic malignant syndrome, inability to adhere to prescribed medication, pregnancy or breastfeeding status, and contraindications for MRI.

This study was reviewed and approved by the Ethics Committee of the Affiliated Mental Health Center of Jiangnan University (Ethical approval number: WXMHCIRB2025LLky004). All participants signed written informed consent forms before the experiment.

### 2.2 Clinical assessment

A comprehensive psychiatric assessment was conducted for all participants by professional psychiatrists. We used PANSS to assess the positive syndromes (PANSS-P), negative syndromes (PANSS-N), and general psychopathology (PANSS-G) of SCZ patients and to identify whether they experience AVH. For AVH patients, we used the Hoffman Auditory Hallucination Rating Scale (HAHRS) to assess the severity of AVH (14). The scale can assess the frequency, reality, loudness, number of voices, length of words, attentional salience, and distress level of AVH. The Modified Overt Aggression Scale (30) and the Buss-Perry Aggression Questionnaire (31) were used to assess participants’ aggressive behavior. The Montreal Cognitive Assessment (32) was used to assess cognitive function. The Hamilton Rating Scale for Anxiety (33), the Hamilton Rating Scale for Depression (34) were used to assess emotional state. The Global Assessment Functioning Scale (35), the Social Disability Screening Schedule (36), and the Perceived Social Support Scale (PSSS) (37)were used to assess global function and social support. Medication doses of participants were calculated using the Olanzapine equivalent dose (38).

### 2.3 MRI data acquisition

Brain imaging was performed using a 3.0-Tesla MRI scanner (MAGNETOM Skyra, Siemens Healthcare, Erlangen, Germany) at the Department of Medical Imaging, Shanghai Health and Medical Center, Wuxi, China. Before scanning, participants were briefed about the procedure to alleviate their anxiety and enhance cooperation. A custom-made sponge pad was used to securely fix the participants’ heads to minimize head motion artifacts in the images. Additionally, sponge earplugs were given to reduce the scanner noise. All participants completed the MRI in an awake state with eyes closed, and no overt signs of hallucinatory behavior (e.g., vocal responses or distressed movements) were observed during the scanning. The specific parameters for the three-dimensional T1-weighted images were as follows: echo time (TE) = 2.98 ms; repetition time (TR) = 2530 ms; flip angle (FA) = 7°; field of view (FOV) = 256×256 mm; slice thickness (ST) = 1.0 mm. The resting-state functional MRI acquisition parameters were as follows: TE = 30 ms, TR = 2000 ms, FA = 90°, FOV = 224×224 mm, ST = 3.5mm; slice number = 33 slices, with a total of 240 time points collected.

### 2.4 MRI data processing

T1-weighted images were processed with the CAT12 toolbox, which is based on SPM12 (https://www.fil.ion.ucl.ac.uk/spm/software/spm12/). The key steps involved in this process are as follows. First, the DICOM files were converted into NIFITI images. Next, spatial normalization was used to align the images with the standard Montreal Neurological Institute template. Then, brain tissues were segmented into white matter, gray matter, and cerebrospinal fluid. The CAT12 toolbox offers a projection-based thickness technique to estimate the central surface of hemispheres in an integrated manner, which is a surface located within the gray matter, approximately midway between the gray-white matter boundary and the pial surface. Then, sulcal depth is calculated based on the Euclidean distance between the central surface and its convex hull (39). Finally, sulcal depth was smoothed using a Gaussian kernel with a full-width at half-maximum (FWHM) of 25 mm (40). To identify and name brain regions, we used the Desikan-Killiany atlas (41). This atlas provides detailed cortical parcellation based on anatomical features. Labels were assigned to cortical regions by matching the central surface with corresponding atlas regions.

Resting-state functional MRI images were processed with the CONN toolbox (https://web.conn-toolbox.org/). Brain regions exhibiting significant differences in sulcal depth were selected as seed regions for FC analysis. The processing steps of FC are briefly described as follows. The first step was to perform head motion correction. Then, spatial normalization was carried out to standardize the data and spatial smoothing using a Gaussian smoothing kernel with 8 mm FWHM (42). Next, the denoising was performed to remove noise from the data. Finally, frequencies below 0.008 Hz and above 0.09 Hz (43) were filtered out to eliminate irrelevant signals. All data underwent quality control, where the MaxMotion values exceeding the threshold of the third quartile plus three times the interquartile range or falling below the first quartile minus three times the interquartile range were excluded as extreme values (44).

### 2.5 Statistical analysis

Statistical analyses were performed using SPSS version 27.0 (IBM Corp., Armonk, NY, USA; http://www.spss.com). Demographic and clinical data were analyzed using independent two-sample t-tests for parametric continuous variables, Mann-Whitney U tests for nonparametric continuous variables, and chi-squared tests for categorical variables such as gender. The threshold for statistical significance was set at *p* < 0.05 (uncorrected).

Group differences in sulcal depth were evaluated by a general linear model (GLM), with age, gender, and years of education as covariates. The significance level was set at a cluster-level corrected *p* < 0.05 using False Discovery Rate (FDR) correction, following an initial vertex-wise threshold of *p* < 0.05. FC between the identified seed regions and all voxels across the whole brain was then evaluated. GLM was employed to compare FC between groups, with age, gender, and years of education included as covariates. The significance level was set at *p* < 0.05, corrected for multiple comparisons using cluster-level FDR correction, following an initial voxel-wise threshold of *p* < 0.05.

Correlation analyses were conducted to examine the relationships between the severity of clinical symptoms (measured by scores on HAHRS, PANSS-P3 hallucination, PANSS-P, PANSS-N, and PANSS-G subscales, PSSS) and both sulcal depth and FC among patients with AVH, controlling for age, gender, and years of education as covariates. For exploratory purposes, the significance threshold for these analyses was set at *p* < 0.05.

## 3 Results

### 3.1 Demographic and clinical characteristics

Demographic and clinical characteristics of the samples are summarized in **Table 1**. A total of 69 participants were enrolled, including 38 AVH patients and 31 NAVH patients. There was missing data for the Olanzapine equivalent dose of antipsychotic medications. Specifically, complete records were available for 26 AVH patients and 20 NAVH patients. The two groups exhibited significant differences in terms of gender and years of education. In AVH patients, there were more females than males. In NAVH patients, there were more males than females. Moreover, AVH patients had a higher level of education in our research. In terms of clinical assessments, significant differences were observed between the two groups in the PANSS-P, PANSS-P3 hallucination, PANSS-N, and PSSS. Compared to NAVH patients, AVH patients exhibited more severe positive symptoms and experienced more intense hallucinations. AVH patients also had fewer negative symptoms and reported higher levels of perceived social support. No statistically significant differences were found in other clinical measures between the groups.

**Table 1:** Demographic and clinical characteristics of two groups.

Variables	AVH (n=38)	NAVH (n=31)	Statistical value	*p*-value
Male/female	17/21	23/8	6.08	0.014^a^
Age (years)	40.03 ± 10.26	41.74 ± 10.59	0.68	0.498^b^
Education (years)	12.00(10.75,13.75)	9.00(8.25,10.75)	2.23	0.026^c^
Duration of illness (years)	15.32 ± 9.58	15.28 ± 8.74	0.02	0.987^b^
PANSS				
PANSS-P	25.00(23.00,26.00)	18.50(15.75,21.25)	5.39	<0.001^c^
PANSS-P3 hallucination	6.00(5.00,6.00)	1.00(1.00,1.00)	7.54	<0.001^c^
PANSS-N	19.50(12.00,24.25)	26.50(23.75,29.00)	2.91	0.004^c^
PANSS-G	38.05 ± 9.09	39.94 ± 7.65	0.92	0.362^b^
PANSS-T	81.58 ± 16.38	82.29 ± 11.96	0.67	0.836^b^
HAHRS	23.16 ± 3.96	–	–	–
MOAS	10.50(0,17.25)	9.00(2.00,21.00)	0.33	0.742^c^
BPAQ	55.00(40.00,70.00)	53.00(30.00,74.50)	0.79	0.428^c^
MoCA	27.00(24.00,28.00)	26.00(21.00,28.00)	0.58	0.559^c^
HAMA	9.76 ± 5.93	10.03 ± 7.40	0.54	0.867^b^
HAMD	7.50(4.00,13.00)	8.00(5.00,13.00)	0.67	0.502^c^
GAF	60.00(55.00,70.00)	62.50(50.00,70.00)	0.50	0.617^c^
SDSS	12.68 ± 4.80	13.77 ± 3.48	0.74	0.222^b^
PSSS	60.34 ± 14.20	49.97 ± 20.64	2.38	0.021^b^
Olanzapine equivalent dose (mg)*	21.28 ± 17.43	19.55 ± 12.41	0.39	0.696 ^b^

The data are expressed as mean ± standard deviation or median (lower quartile, upper quartile); ^a^, chi-squared test; ^b^, two-sample t-test; ^c^, Mann-Whitney U-test; AVH, schizophrenia patients with auditory verbal hallucinations; NAVH, schizophrenia patients without auditory verbal hallucinations; PANSS, Positive and Negative Syndrome Scale; PANSS-P, positive syndromes of PANSS; PANSS-N, negative syndromes of PANSS; PANSS-G, general psychopathology of PANSS; PANSS-T, total score of PANSS; HAHRS, Hoffman Auditory Hallucination Rating Scale; MOAS, Modified Overt Aggression Scale; BPAQ, Buss-Perry Aggression Questionnaire; MoCA, Montreal Cognitive Assessment; HAMA, Hamilton Rating Scale for Anxiety; HAMD, Hamilton Rating Scale for Depression; PANAS, Positive and Negative Affect Schedule; GAF, Global Assessment Functioning Scale; SDSS, Social Disability Screening Schedule; PSSS, Perceived Social Support Scale; SES, Rosenberg Self-Esteem Scale; *, For Olanzapine equivalent dose, effective sample size in AVH is 26 patients, and in NAVH is 22 patients.

### 3.2 Differences in sulcal depth between groups

**Figure 1:**
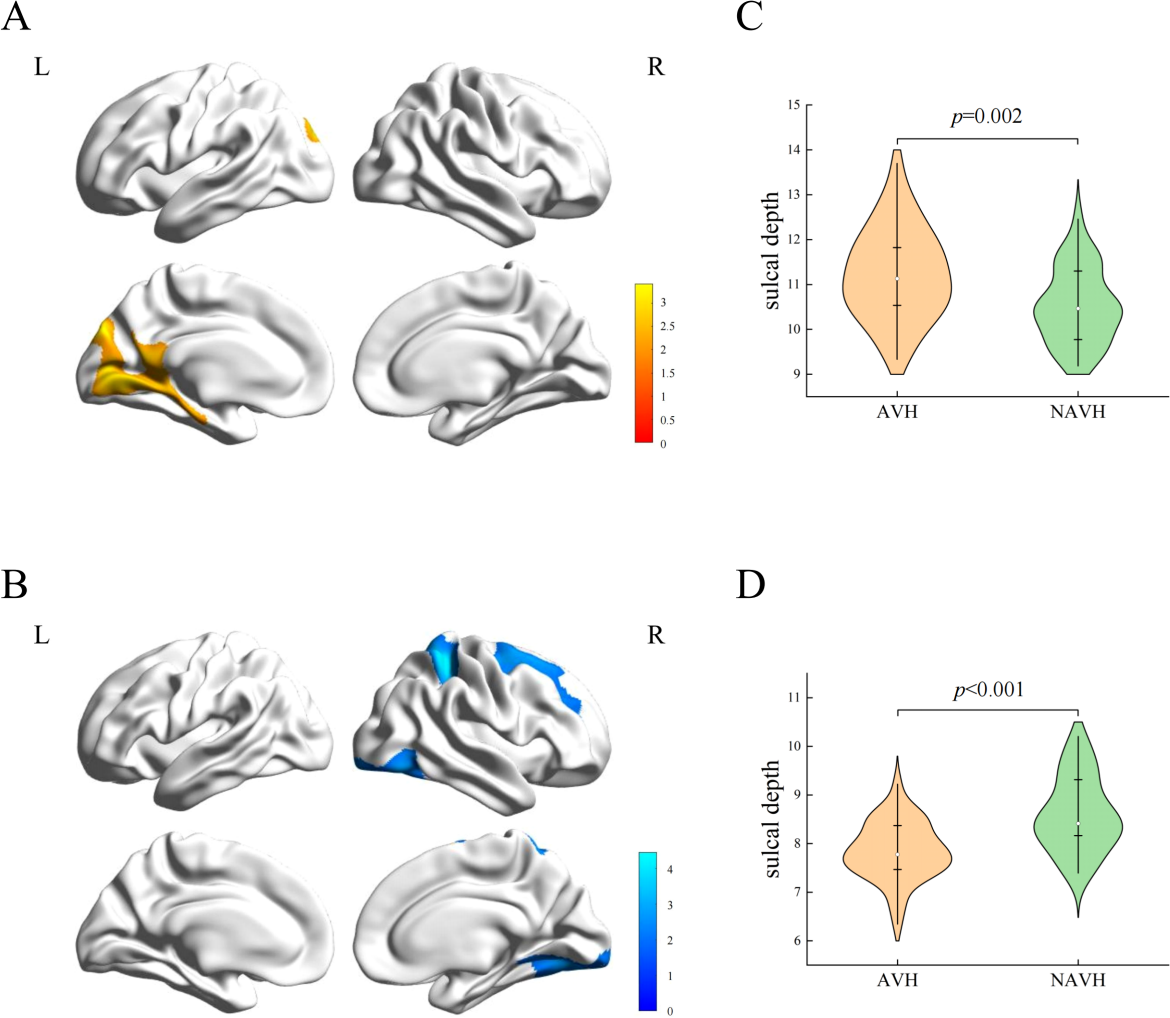
Sulcal depth differences between two groups. **(A)** The cluster of increased sulcal depth in AVH patients compared to NAVH patients. **(B)** The cluster of decreased sulcal depth in AVH patients compared to NAVH patients. **(C)** The difference in the mean values of the increased cluster between the two groups. **(D)** the difference in the mean values of the decreased clusters between the two groups.

Compared with NAVH patients, AVH patients exhibited a cluster of increased sulcal depth in the left hemisphere, including the lingual gyrus, cingulate gyrus, pericalcarine cortex, parahippocampal gyrus (PHG), SPG, cuneus (CUN), and precuneus (PCUN) (**Table 2** and **Figure 1A**). The mean values of the cluster between the two groups were significantly different (**Figure 1C**). Besides, AVH patients showed three clusters of decreased sulcal depth in the right hemisphere, particularly in the SPG, SFG, lingual gyrus, lateral occipital cortex, fusiform gyrus, postcentral gyrus, middle frontal gyrus (MFG), precentral gyrus (PreCG), ITG, PCUN, and PHG (**Table 2** and **Figure 1B**). The mean values of the cluster between the two groups were significantly different (**Figure 1D**).

**Table 2:** Brain regions show sulcal depth difference between two groups.

Cluster	sulcal depth	Hemisphere	Cluster size	Brain Region	MNI coordinate			*t*-value
					*x*	*y*	*z*	
1	AVH>NAVH	L	1662	lingual gyrus	-2	-61	5	3.08
				cingulate gyrus	-2	-50	16	2.71
				CAL	-9	-73	12	3.02
				PHG	-14	-42	1	2.53
				SPG	2	-75	29	3.35
				cuneus	2	-72	27	3.42
				precuneus	-2	-53	16	2.77
2	AVH<NAVH	R	1200	lingual gyrus	18	-64	4	3.02
				LOC	43	-57	-10	2.94
				fusiform gyrus	18	-63	-4	2.94
				ITG	50	-52	-8	2.62
				PHG	23	-38	0	2.31
3	AVH<NAVH	R	1083	SFG	13	-25	70	2.85
				MFG	24	9	60	2.68
				precentral gyrus	15	-27	69	2.73
4	AVH<NAVH	R	959	SPG	33	-53	59	4.46
				postcentral gyrus	31	-48	58	3.99
				precuneus	15	-68	54	2.66

AVH, schizophrenia patients with auditory verbal hallucinations; NAVH, schizophrenia patients without auditory verbal hallucinations; L, left; R, right; MNI, Montreal Neurological Institute; CAL, pericalcarine cortex; PHG, parahippocampal gyrus; SPG, superior parietal gyrus; LOC, lateral occipital cortex; ITG, inferior temporal gyrus; SFG, superior frontal gyrus; MFG, middle frontal gyrus.

### 3.3 FC abnormalities associated with differences in sulcal depth

**Figure 2:**
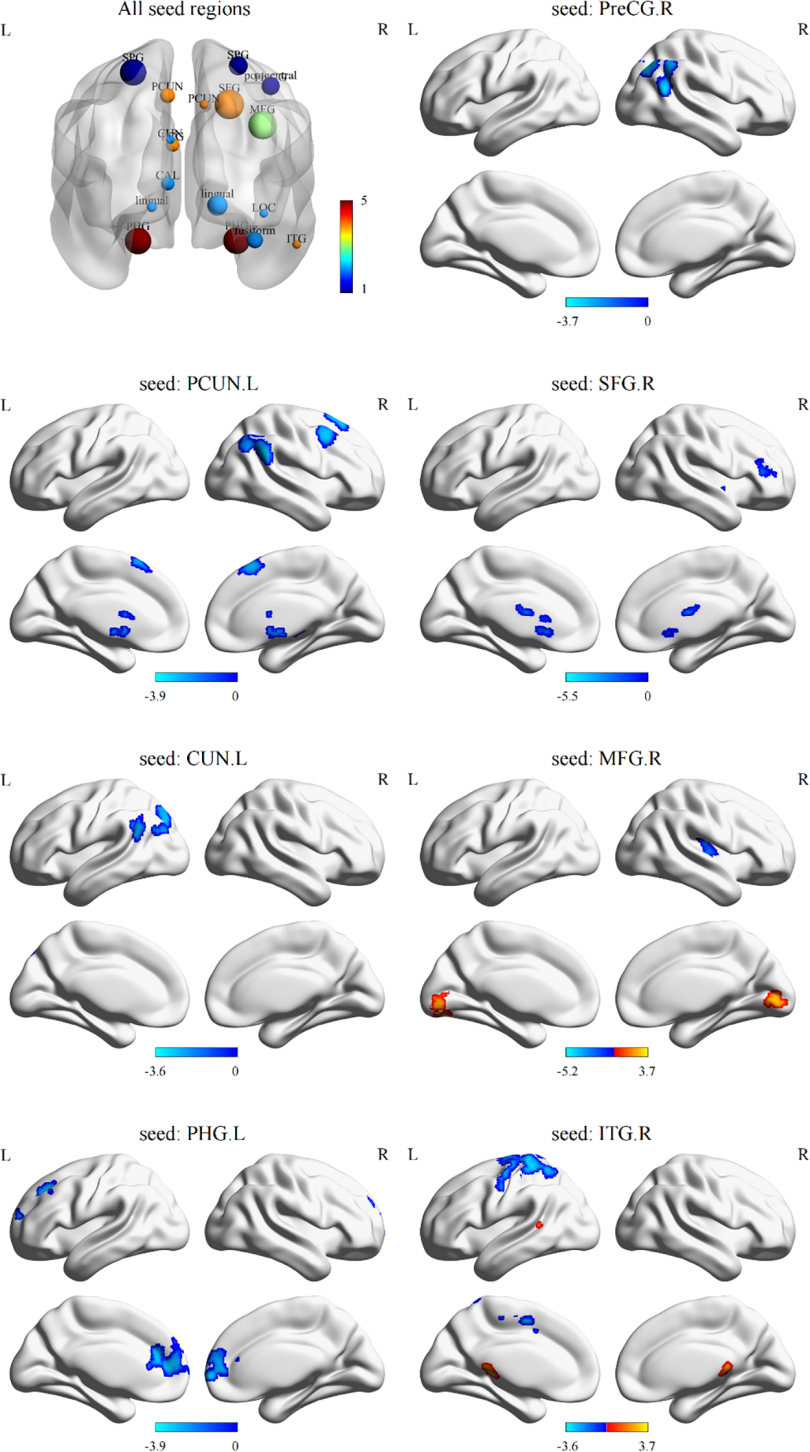
Differences in functional connectivity using sulcal depth differences as seed regions between two groups. All seed regions refer to the eighteen seed regions selected based on the brain regions with differences in sulcal depth. Among them, the following seven seed regions show abnormal functional connectivity. L, left hemisphere; R, right hemisphere; SPG, superior parietal gyrus; PCUN, precuneus; PreCG, precentral gyrus; SFG, superior frontal gyrus; MFG, middle frontal gyrus; CUN, cuneus; PCG, posterior cingulate gyrus; CAL, pericalcarine cortex; LOC, lateral occipital gyrus; PHG, parahippocampal gyrus; ITG, inferior temporal gyrus.

Based on sulcal depth results, brain regions with sulcal depth differences were defined as seed regions. We selected 18 seed regions (**Figure 2**, All seed regions), of which 7 seed regions exhibited FC abnormalities (**Table 3**; **Figure 2**), including left PHG, left CUN, left PCUN, right SFG, right MFG, right PreCG, and right ITG. FC analysis revealed widespread connectivity reductions in AVH patients compared to NAVH patients. Specifically, we observed reduced FC between left PHG and right SFG and left caudate, between left CUN and left lateral occipital cortex, between left PCUN and left putamen, right SFG, right angular gyrus, between right SFG and right putamen, between right MFG and right putamen, between right PreCG and right lateral occipital cortex, between right ITG and left postcentral gyrus. Conversely, increased FC was identified between right ITG and brainstem, as well as between the right MFG and left calcarine cortex.

**Table 3:** Brain regions show alterations of FC between two groups.

Brain Region	FC	Hemisphere	Cluster size	MNI coordinate			*p*-FDR*
				*x*	*y*	*z*	
Seed: PCUN.L							
Putamen	AVH<NAVH	L	2500	-14	-2	+4	<0.001
SFG	AVH<NAVH	R	1546	+14	+24	+54	0.003
Cerebelum	AVH<NAVH	L	988	-14	-62	-30	0.029
Angular Gyrus	AVH<NAVH	R	908	+50	-54	+54	0.033
Seed: CUN.L							
LOC	AVH<NAVH	L	1173	-32	-54	+24	0.044
Seed: PHG.L							
SFG	AVH<NAVH	R	1942	+14	+54	+12	0.001
Caudate	AVH<NAVH	L	1194	-18	+6	+18	0.017
Seed : PreCG.R							
LOC	AVH<NAVH	R	1284	+32	-80	+48	0.044
Seed: SFG.R							
Putamen	AVH<NAVH	R	4788	+22	+24	-2	<0.001
Seed: MFG.R							
Putamen	AVH<NAVH	R	2057	+22	+26	-2	0.001
Calcarine Cortex	AVH>NAVH	L	1082	-16	-78	+6	0.040
Seed: ITG.R							
Brainstem	AVH>NAVH	L	1360	-8	-34	+20	0.020
Postcentral Gyrus	AVH<NAVH	L	1293	-24	-36	+74	0.020

FC, functional connectivity; MNI, Montreal Neurological Institute; *, cluster size corrected *p* < 0.05 False Discovery Rate after applying a voxel threshold of *p* < 0.05; AVH, schizophrenia patients with auditory verbal hallucinations; NAVH, schizophrenia patients without auditory verbal hallucinations; L, left; R, right; PCUN, precuneus; SFG, superior frontal gyrus; CUN, cuneus; LOC, lateral occipital cortex; PHG, parahippocampal gyrus; PreCG, precentral gyrus; MFG, middle frontal gyrus; ITG, inferior temporal gyrus.

### 3.4 Correlation of sulcal depth and FC with psychiatric symptoms

**Figure 3:**
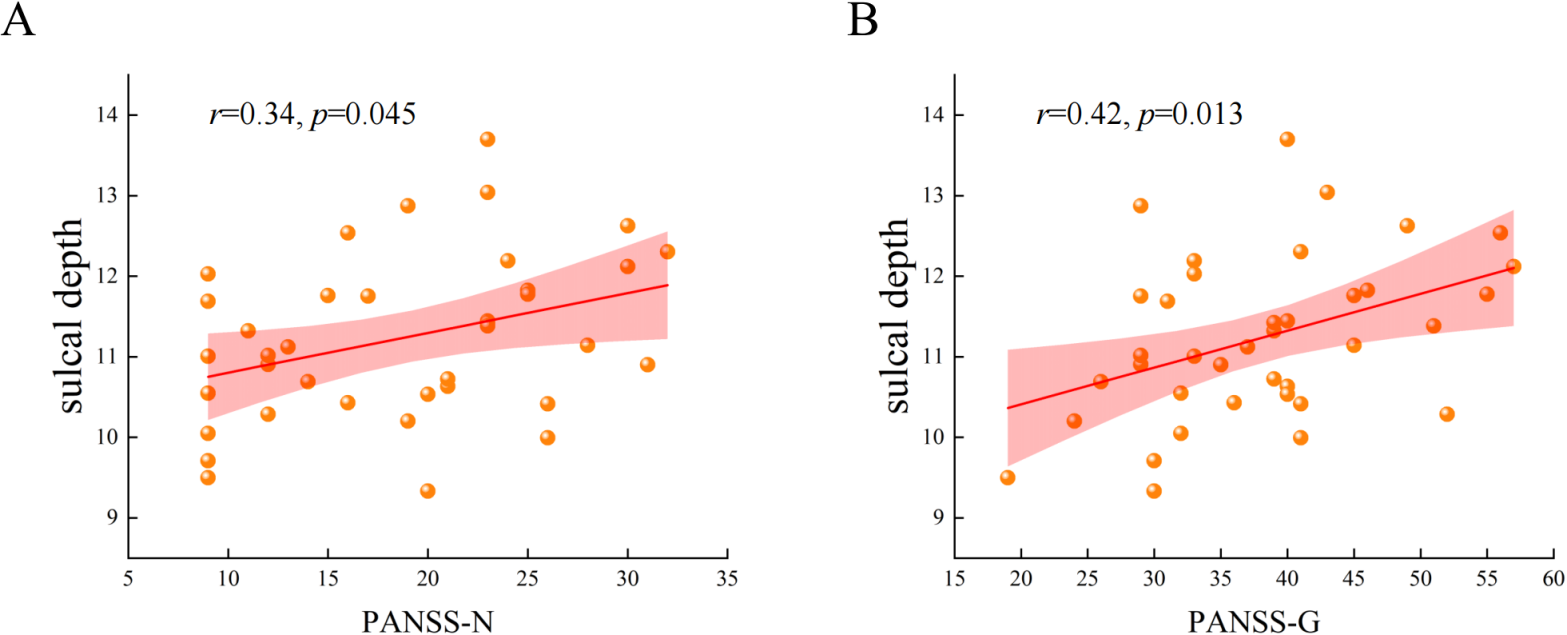
The correlation between the mean values of the increased cluster in sulcal depth with PANSS-N and PANSS-G in AVH patients. **(A)** The mean values of the increased cluster in sulcal depth were positively correlated with the scores of negative syndromes of Positive and Negative Syndrome Scale. **(B)** The mean values of the increased cluster in sulcal depth were positively correlated with the scores of general psychopathologies of Positive and Negative Syndrome Scale.

The correlation analysis showed that the mean value of the increased sulcal depth cluster in AVH patients was positively correlated with the PANSS-N (*r* = 0.34, *p* = 0.045, uncorrected) (**Figure 3A**). This cluster also exhibited a significant positive correlation with the PANSS-G (*r* = 0.42, *p* = 0.013, uncorrected) (**Figure 3B**). The specific results regarding the correlation of sulcal depth and FC with psychiatric symptoms are presented in the **Supplementary Materials** (**Supplementary Table S1**). Regrettably, these results did not survive multiple comparison correction.

## 4 Discussion

This study analyzed the sulcal depth and associated FC in brain regions of AVH patients and NAVH patients and drew three conclusions. First, the cluster of increased sulcal depth in AVH patients was located in the left hemisphere, including the lingual gyrus, cingulate gyrus, pericalcarine cortex, PHG, SPG, CUN, and PCUN. Then, the clusters of decreased sulcal depth were observed in the right hemisphere, including SPG, SFG, lingual gyrus, lateral occipital cortex, fusiform gyrus, postcentral gyrus, MFG, PreCG, ITG, PCUN, and PHG. Secondly, our seed-based FC analysis revealed significantly decreased FC between the seed regions and several areas, such as the SFG, angular gyrus, putamen, and others. Third, in AVH patients, the brain regions with increased sulcal depth cluster were significantly correlated with PANSS-N and PANSS-G.

Our study identified abnormal sulcal depth in key regions of the default mode network (DMN), such as the PCUN, cingulate gyrus, and SPG, and found abnormal FC when using the PCUN as seed regions. DMN plays an important role in self-reference, social cognition, episodic and autobiographical memory, language and semantic memory, and mind wandering (45). Structurally, we observed abnormal sulcal depth alterations in key DMN regions, consistent with findings from Katharina et al. (46) and Shen et al. (14), who reported elevated local gyrification index in the PCUN, cingulate gyrus, and SPG. These structural changes may influence the pathogenesis of AVH in two critical ways. First, they impair spatial information processing and disrupt sensory integration. Second, they weaken self-referential processing, thereby exacerbating patients’ misattribution of hallucinatory sources (45, 47). Functionally, our study revealed three key connectivity abnormalities. First, the diminished FC from PCUN to putamen resonated with previous findings of causal disconnections in the striatal-DMN circuit (48), underscoring the crucial role of the striatal-DMN circuit in AVH neurobiology. Second, reduced FC between PCUN and angular gyrus reflects dysfunction in the lateral parietal system, which is a hub for internal speech monitoring and self-referential integration, suggesting impaired mechanisms for monitoring internally generated speech (49, 50). Third, the reduced FC between the PCUN and SFG in AVH patients may indicate that disruption in the crucial interplay and top-down modulation between the DMN and cognitive control network is disrupted. Xie et al. (51) found that low-frequency repetitive transcranial magnetic stimulation can improve the FC between these two regions in AVH patients and alleviate hallucination symptoms, further confirming their significant role in AVH.

Additionally, a notable reduction in FC was exhibited between the putamen and brain regions such as the SFG and MFG. Putamen is a key component of the striatum, the reduced FC may impair the striatum’s role in filtering and monitoring spontaneous activations. This phenomenon is consistent with the theory that cortico-striatal dysfunction disrupts gating mechanisms, allowing unmodulated internal signals to enter consciousness as hallucinations (52). Moreover, hallucinations are mediated by increased dopamine levels within the striatum (53), which is also an important target for antipsychotic medications. Chen et al. (54) proposed a significant correlation between the putamen-auditory cortex connectivity and the neuropathological mechanisms of AVH. Yang et al. also confirmed that the FC of prefrontal-striatal plays an important role in attention and executive dysfunction in SCZ patients (55). Our study further elucidates the significant role of the prefrontal-striatal neural circuits in the neuropathology of AVH.

We also found a significant decrease in sulcal depth in the PHG, which is anatomically connected to the cingulate gyrus, temporal lobe, and prefrontal cortex. PHG is a key component of the Papez circuit, which is a circuit believed to control memory, emotions, and motivations (56, 57). In addition, the PHG is associated with auditory working memory and related to the negative emotions associated with tinnitus (58, 59). A review (60) proposed that the PHG relays AVH memories to the auditory cortex to consolidate acoustic representations and maintain perceptual memory, which is mistakenly categorized as external stimuli. These findings support the theory the memory deficit model of source-monitoring problems (52). PHG is a key region within the medial temporal lobes, playing a crucial role in both relational encoding and memory retrieval. Damage or dysfunction in the PHG may lead to impaired binding and retrieval of memory features, thereby affecting an individual’s ability to accurately judge the source of memories (61). Moreover, reduced FC between PHG and SFG directly implicates dysfunctional prefrontal-limbic regulation. This may impair top-down modulation over memory integration processes, potentiating source monitoring failures through misattribution of internal representations.

In the study of cortical structure in AVH patients, we observed changes in sulcal depth in brain regions such as the lingual gyrus, pericalcarine cortex, lateral occipital cortex, fusiform gyrus, and CUN. These regions are primarily responsible for integrating and processing visual information. Previous studies reported that patients with AVH exhibit similarly aberrant FC to multiple brain regions when seeded in the auditory cortex. A widely accepted hypothesis suggested that disrupted FC between visual processing regions (occipital cortex) and auditory neural circuits may induce dysregulated phonological semantic integration mechanisms, potentially serving as a neurobiological substrate for the emergence of AVH (62). Other studies found reduced low-frequency fluctuation amplitude in the lingual gyrus and occipital cortex in SCZ patients (63), and hypoperfusion in the calcarine cortex in AVH patients (64). Lewis et al. (65) proposed that audiovisual synchrony enhances the connection between early visual and auditory areas to improve motion discrimination. Although AVH is classified as an auditory perceptual abnormality, the sensory systems of the brain do not operate independently. Changes in visual-related brain regions may disrupt the integration of perceptual information, thereby impairing normal auditory perception and leading to the occurrence of AVH.

The ITG is also a crucial part of the temporal lobe cortex and belongs to the ventral visual pathway. Although its primary functions are related to vision, it also collaborates with the auditory cortex during language processing, playing a significant role in multi-channel sensory integration, language, and semantic memory (66). Changes in its sulcal depth may affect the feature analysis and pattern recognition of auditory stimuli, leading to deviations in the brain’s processing of normal auditory information. This can cause the brain to misidentify internally generated abnormal signals as real sounds, triggering AVH. A meta-analysis found that SCZ patients generally have cortical thinning in the right temporal lobe (67). Xie et al. (64) reported increased cerebral blood flow in the left ITG and bilateral putamen in AVH patients. Additionally, our study discovered that in AVH patients, FC between the ITG and the brainstem is enhanced, while FC between the ITG and the postcentral gyrus is weakened. Previous studies reported that the increased FC between the ITG and the brainstem in AVH patients may be related to excessive bottom-up signal activation (68). The postcentral gyrus, a brain region associated with inner speech, is fundamental to its generation through its neural activity. Together with the PreCG, it is activated during AVH experiences and plays a significant role in processing and transmitting sensorimotor information (52). The reduced FC between the ITG and postcentral gyrus suggested impaired internal speech monitoring, hindering the verification of auditory perception authenticity and ultimately exacerbating AVH experiences (69, 70).

It is worth noting that our study found that the increased sulcal depth cluster in AVH patients (including the left cingulate gyrus, SPG, PCUN, PHG, etc.) was positively correlated with the PANSS-N and PANSS-G. This may suggest that in AVH patients, the cingulate gyrus is associated with negative symptoms such as apathy, the PHG is related to memory impairments and cognitive dysfunction, and the SPG may be linked to attention deficits and abnormalities in sensory information processing. This study unexpectedly found no significant correlation between the severity of AVH and the sulcal depth or FC. From a neurobiological mechanism perspective, FC changes might more likely reflect the transient neural state of AVH rather than the severity of chronic symptoms (71). Additionally, most AVH patients also present with other symptoms, such as delusions, a certain degree of confusion, and negative symptoms, this result may be related to the diversity of symptoms (72).

This study has several limitations that should be acknowledged. First, a significant limitation is the gender disparity between two groups, with a higher proportion of females in the AVH patients. This gender imbalance may reflect differences in disease characteristics or recruitment bias. Additionally, there was a slight mismatch in years of education between the two groups, which could potentially influence our findings. Although we included gender and years of education as covariates in our regression models, residual confounding may still exist. Future studies should aim to use more matched samples and conduct stratified analyses to explore whether gender or years of education independently influence AVH expression. Second, our study lacked healthy controls, limiting our ability to determine if the observed differences are specific to AVH or general to SCZ. However, our design aimed to explore the neural mechanisms underlying the AVH subtype within SCZ, shedding some light on potential biological correlations of AVH-related sulcal depth and FC through comparisons between the two SCZ groups. Future research should include diverse populations to better clarify specificity. Third, despite a series of analyses conducted, due to missing data on antipsychotic dosages, the potential confounding effects of antipsychotic medications on the study metrics have not been fully ruled out. In future studies, we plan to systematically record detailed antipsychotic dosage information or recruit more drug-naive patients with first-episode illnesses. Fourth, a key limitation is the inability to confirm whether participants were experiencing active AVH during scanning, which may have influenced the interpretation of FC alterations as either state or trait related. Despite these limitations, our study holds significant clinical and scientific value. This is the first research to utilize multimodal brain imaging techniques for demonstrating abnormal sulcal depth and associated FC abnormalities in SCZ patients with AVH.

## 5 Conclusions

In conclusion, this study is the first to reveal abnormalities in sulcal depth and associated FC abnormalities in SCZ patients with AVH, providing neuroimaging evidence for understanding the neural mechanisms underlying AVH. Our findings suggest that these abnormalities may originate early in neurodevelopment and involve key brain regions and networks, including the DMN, prefrontal-striatal pathway, visual cortex, and the limbic system. These systems may interact to disrupt self-monitoring and sensory processing, thereby contributing to the emergence of AVH. Sulcal depth may serve as a potential imaging biomarker for the early identification of individuals at risk for AVH. However, the utility of sulcal depth for guiding targeted neuromodulation, such as low-frequency repetitive transcranial magnetic stimulation, remains to be determined. While our whole-brain findings suggest broader network involvement beyond established targets, translating this into novel interventions requires future evidence demonstrating that sulcal morphology is modifiable and functionally relevant to outcomes. Our results thus provide a preliminary reference for exploring broader targets, meriting further investigation.

## Data Availability

The raw data supporting the conclusions of this article will be made available by the authors without undue reservation.

## Supplementary material

Supplementary Table 1 Correlation of sulcal depth and FC with psychiatric symptoms in AVH patients.
