# Supplementary material for: Alterations in sulcal depth and associated functional connectivity in schizophrenia with auditory verbal hallucinations
Source: Front Psychiatry. 2025 Jul 30;16:1641190. doi: 10.3389/fpsyt.2025.1641190 (PMC12343575; doi:10.3389/fpsyt.2025.1641190)
Supplement: Supplementary Table 1 — Correlation of sulcal depth and FC with psychiatric symptoms in AVH patients. [file Table1.docx]

**Supplementary Materials**

| Table S1. Correlation of sulcal depth and FC with psychiatric symptoms in AVH patients. | | | | | | | | |
| --- | --- | --- | --- | --- | --- | --- | --- | --- |
| Variable | Measure | HAHRS | PANSS-P3  hallucination | PANSS-P | PANSS-N | PANSS-G | PSSS | Olanzapine equivalent  dose (mg)* |
| increased sulcal depth | r | 0.01 | -0.10 | -0.06 | 0.34 | 0.42 | 0.11 | -0.08 |
|  | *p*-value | 0.939 | 0.572 | 0.748 | 0.045 | 0.013 | 0.521 | 0.652 |
| decreased sulcal depth | r | -0.18 | -0.23 | -0.23 | 0.04 | -0.11 | 0.06 | -0.15 |
|  | *p*-value | 0.293 | 0.185 | 0.193 | 0.815 | 0.544 | 0.726 | 0.389 |
| seed: PCUN.L decreased FC | r | 0.13 | 0.29 | -0.09 | 0.10 | 0.25 | -0.19 | -0.09 |
|  | *p*-value | 0.473 | 0.102 | 0.636 | 0.580 | 0.176 | 0.295 | 0.594 |
| seed: CUN.L decreased FC | r | -0.11 | 0.27 | -0.02 | 0.03 | -0.12 | -0.23 | -0.01 |
|  | *p*-value | 0.547 | 0.135 | 0.906 | 0.859 | 0.518 | 0.234 | 0.994 |
| seed: PHG.L decreased FC | r | 0.25 | 0.10 | 0.03 | 0.03 | 0.26 | 0.00 | -0.08 |
|  | *p*-value | 0.171 | 0.575 | 0.889 | 0.870 | 0.148 | 0.982 | 0.605 |
| seed: PreCG.R decreased FC | r | -0.10 | -0.35 | -0.04 | 0.10 | 0.22 | 0.15 | -0.27 |
|  | *p*-value | 0.598 | 0.052 | 0.821 | 0.584 | 0.234 | 0.419 | 0.093 |
| seed: SFG.R decreased FC | r | -0.20 | -0.27 | 0.00 | -0.03 | 0.19 | 0.08 | -0.15 |
|  | *p*-value | 0.272 | 0.141 | 0.994 | 0.876 | 0.310 | 0.679 | 0.350 |
| seed: MFG.R decreased FC | r | -0.22 | -0.04 | 0.03 | 0.00 | 0.23 | 0.15 | -0.07 |
|  | *p*-value | 0.234 | 0.832 | 0.852 | 0.983 | 0.200 | 0.429 | 0.669 |
| seed: MFG.R increased FC | r | 0.01 | -0.08 | 0.08 | -0.13 | 0.03 | 0.16 | 0.25 |
|  | *p*-value | 0.977 | 0.666 | 0.665 | 0.474 | 0.855 | 0.376 | 0.118 |
| seed: ITG.R increased FC | r | -0.34 | -0.15 | -0.04 | 0.04 | -0.14 | -0.07 | 0.28 |
|  | *p*-value | 0.060 | 0.402 | 0.810 | 0.841 | 0.463 | 0.699 | 0.077 |
| seed: ITG.R decreased FC | r | 0.03 | -0.13 | -0.10 | 0.10 | -0.10 | 0.29 | -0.17 |
|  | *p*-value | 0.873 | 0.472 | 0.576 | 0.593 | 0.573 | 0.112 | 0.276 |
| *: For Olanzapine equivalent dose, effective sample size in AVH is 26 patients. FC: functional connectivity; AVH: schizophrenia patients with auditory verbal hallucinations; PANSS: Positive and Negative Syndrome Scale; PANSS-P: positive syndromes of PANSS, PANSS-N: negative syndromes of PANSS; PANSS-G: general psychopathology of PANSS; HAHRS: Hoffman Auditory Hallucination Rating Scale; ; PSSS: Perceived Social Support Scale; L: left; R: right; PCUN: precuneus; CUN: cuneus; PHG: parahippocampal gyrus; PreCG: precentral gyrus; SFG: superior frontal gyrus; MFG: middle frontal gyrus; ITG: inferior temporal gyrus. | | | | | | | | |
